# Impact of Sepsis on the Oncologic Outcomes of Advanced Epithelial Ovarian Cancer Patients: A Multicenter Observational Study

**DOI:** 10.3390/cancers15184642

**Published:** 2023-09-20

**Authors:** Sherin A. Said, Joanne A. de Hullu, Maaike A. van der Aa, Janneke E. W. Walraven, Ruud L. M. Bekkers, Brigitte F. M. Slangen, Peter Pickkers, Anne M. van Altena

**Affiliations:** 1 Department of Obstetrics and Gynecology, Radboud University Medical Center, 6525 EP Nijmegen, The Netherlands; joanne.dehullu@radboudumc.nl (J.A.d.H.); anne.vanaltena@radboudumc.nl (A.M.v.A.); 2 Department of Research and Development, Netherlands Comprehensive Cancer Organization (IKNL), 3511 DT Utrecht, The Netherlands; m.vanderaa@iknl.nl; 3 Department of Medical Oncology, Radboud University Medical Center, 6525 EP Nijmegen, The Netherlands; 4 Department of Obstetrics and Gynecology, Catharina Hospital, 5623 EJ Eindhoven, The Netherlands; 5 GROW–School for Oncology and Reproduction, University of Maastricht, 6229 GT Maastricht, The Netherlands; 6 Department of Obstetrics and Gynecology, Maastricht University Medical Centre, 6229 HX Maastricht, The Netherlands; 7 Department of Intensive Care Medicine, Radboud University Medical Center, 6525 EP Nijmegen, The Netherlands; peter.pickkers@radboudumc.nl

**Keywords:** epithelial ovarian cancer, immune system, sepsis, survival, immune response

## Abstract

**Simple Summary:**

There has been a growing interest in the interplay between the immune system and the prognosis of epithelial ovarian cancer (EOC). It has become clear that EOC has the ability to escape destruction by the immune system by creating a highly immunosuppressive microenvironment in the abdominal cavity. The first case of an advanced-stage EOC patient who experienced the complete disappearance of her cancer following sepsis without undergoing cancer treatment was reported in 2018. Sepsis has the ability to both activate and suppress the immune system, presenting potential beneficial as well as detrimental effects. It is still unclear what impact sepsis has on the prognosis of advanced-stage EOC. This study’s aim was to assess the impact of sepsis on the oncologic outcomes of advanced-stage EOC patients. Of 215 OC patients, a total of 18 advanced-stage EOC patients experienced sepsis. Their survival outcomes were compared with 3988 unmatched and 54 matched patients from the Netherlands Cancer Registry (NCR). The overall and progression-free survival was similar between the sepsis and (un)matched patients from the NCR. Our study suggests that sepsis and its subsequent immune response, overall, does not substantially influence the prognosis of EOC patients.

**Abstract:**

Objective: The sepsis-induced inflammatory response may potentially affect malignant cells. Recently, a case of spontaneous regression of a histologically confirmed International Federation of Gynecology and Obstetrics (FIGO) stage IIIC epithelial ovarian cancer (EOC) following sepsis was reported. The aim of our study was to assess the impact of sepsis on the oncologic outcomes of advanced-stage EOC patients. Methods: Gynecologic oncologic patients admitted to the Intensive Care Unit of three oncologic centers between 2006 and 2019 were identified and patients who experienced sepsis following advanced-stage EOC diagnosis were selected. Survival outcomes were compared with advanced-stage EOC patients from the Netherlands Cancer Registry (NCR). To correct for case-mix differences, propensity score matching using 1:3 nearest neighbor matching was conducted after which survival analyses were repeated. Results: A total of 18 of 215 patients with advanced-stage EOC experienced sepsis. Sepsis patients had similar distributions of patient, tumor, and treatment characteristics to 3988 patients from the NCR cohort. A total of 3 of 18 patients died from the complications of sepsis. While the remaining patients initially responded to treatment, 14/15 patients relapsed. The median (IQR) overall survival was 31 (24–44) and 35 (20–60) months for the sepsis and unmatched NCR cohort (*p* = 0.56), respectively. The median (IQR) progression-free survival was 16 (11–21) and 16 (11–27) months (*p* = 0.90), respectively. Survival outcomes did not differ following propensity matching (overall survival of 31 (24–44) vs. 36 (20–56) months, *p* = 0.40; progression-free survival of 16 (11–21) and 16 (12–21) months, *p* = 0.72). Conclusion: In this observational study, the occurrence of sepsis did not affect the oncologic and survival outcomes of advanced-stage EOC patients.

## 1. Introduction 

The vast majority of patients with epithelial ovarian cancer (EOC) are diagnosed at an advanced stage [1]. Despite enhancements in treatment strategies, such as more radical surgery, combination chemotherapy, intraperitoneal chemotherapy, or targeted molecular therapy, the long-term survival of advanced-stage EOC patients has only improved slightly over the past three decades [1,2,3,4]. Hence, EOC remains the leading cause of gynecologic cancer-related death in the Western world [5]. The role of the immune system in ovarian cancer has been an important focus of research [1,4,6,7]. It has become clear that instead of being targeted for immune destruction, ovarian cancer has the ability to escape the immune system [4,8,9]. The foremost mechanism behind this evasion of immunosurveillance is the creation of a highly immunosuppressive environment in the peritoneal cavity [4,8,9]. The immunologic response against ovarian cancer is a critical balance between immune-activating and immune-suppressing mechanisms [1,4,7,9,10,11,12].

Of interest, the first case report of spontaneous regression of advanced-stage EOC following sepsis illustrates the interplay between immunity and cancer prognosis [13]. Briefly, a 79-year-old patient developed sepsis due to a bowel perforation after a biopsy, confirming a high-grade serous FIGO stage IIIC EOC diagnosis. She was admitted to the Intensive Care Unit (ICU) and treated for her sepsis. She was later discharged with supportive care. In the following six months, she showed no signs of EOC. She underwent an uncomplicated bilateral salpingo-oophorectomy after which histopathological examination only confirmed a microscopic residual tumor in one ovary. To date, seven years after diagnosis, without undergoing any adjuvant systemic chemotherapy, the patient shows no signs of recurrent disease.

Spontaneous regression of cancer is defined as the partial or complete disappearance of a histologically proven malignant tumor, either in the absence of treatment or in the presence of therapy considered inadequate to exert a significant influence on the disease [14]. This phenomenon has been reported for cancers such as acute myeloid leukemia, lymphoma, melanoma, and sarcoma, but not for EOC prior to the above-mentioned case [15,16,17,18]. Spontaneous regression of cancer has mostly been observed following infections (including sepsis) with various pathogens [14]. It is hypothesized that the systemic inflammatory response, triggered by sepsis, could elicit an antitumor response, which can lead to favorable oncologic outcomes. However, in theory, it is also possible that a persistent immunosuppressive response, observed in sepsis patients, may lead to unfavorable oncologic outcomes [17]. Therefore, the aim of this pilot study was to assess the impact of sepsis on the course and oncologic outcomes of advanced-stage EOC patients.

## 2. Methods

### 2.1. Study Population

To identify advanced-stage EOC patients who experienced sepsis, gynecologic oncologic patients admitted to the ICU between 1 January 2006 and 1 January 2019 (to allow a last follow-up date of 31 January 2022) were selected from the following three Dutch hospitals: Radboud University Medical Center, Catharina Hospital, and Maastricht University Medical Center. Patients were identified from the hospitals’ electronic patient records using the search terms ‘neoplasm’ and ‘gynecology as the referring medical specialty’. Subsequently, patients were cross-checked with a list of all consecutive ovarian cancer patients who underwent treatment in the participating hospitals. Moreover, the study’s eligibility criteria required patients:

1. To be diagnosed with a histologically confirmed EOC;
2. To have FIGO stage IIB or higher (i.e., advanced-stage EOC);
3. To be admitted to the ICU;
4. To be diagnosed with sepsis [19,20] following their primary or recurrent EOC diagnosis;
5. To have an abdominal focus for their sepsis.

Patients who did not meet all of the above-mentioned criteria were excluded from this study.

In addition, data on advanced-stage EOC patients was obtained from the Netherlands Cancer Registry (NCR) to gain insights into the survival of advanced-stage EOC patients in general and to compare survival outcomes with the sepsis patients identified in this study. The NCR is a nationwide population-based registry that is notified weekly of all newly histologically confirmed malignancies in the Netherlands through an automated nationwide pathology archive (PALGA). Dedicated registrars previously collected data on patient, tumor, and treatment characteristics from patients’ medical records. No additional data were collected for the NCR cohort in this study. Further details on the NCR and the nationwide cohort used in the survival analyses were reported earlier [21].

### 2.2. Definitions

Sepsis was defined according to the Sepsis-2 (introduced in 2001) or Sepsis-3 (introduced in 2016) definition, as the criteria of sepsis changed throughout the period in which data were collected for this study [19,20]. According to the Sepsis-2 definition, sepsis is defined as a proven or suspected infection accompanied by at least two Systemic Inflammatory Response Syndrome (SIRS) criteria (see Supplementary Table S1) [19]. According to the Sepsis-3 definition, sepsis is defined as organ dysfunction caused by a dysregulated host response to an infection. Organ dysfunction is here characterized by an increase in Sequential Organ Failure Assessment (SOFA) score of 2 or greater (see supplementary Table S2) [20]. The severity of sepsis was assessed on the requirement of a vasopressor to maintain an adequate mean arterial pressure (i.e., vasopressor-dependent sepsis or non-vasopressor-dependent sepsis). Septic shock was defined as a subset of sepsis in which particularly profound circulatory, cellular, and metabolic abnormalities were associated with a greater risk of mortality than with sepsis alone [20]. These patients are characterized by the need for a vasopressor, as well as an elevated lactate level.

### 2.3. Data Collection

Data were collected from patients’ medical records for the sepsis cohort. The collected data included information regarding the EOC diagnosis and treatment, e.g., age at diagnosis, FIGO stage, histologic subtype, treatment approach, surgical procedures performed during cytoreductive surgery, residual disease after cytoreductive surgery, hospital length of stay, chemotherapy regimen, and EOC treatment response. In addition, collected data included information on the sepsis diagnosis and treatment, e.g., site of infection, the time between the date of cytoreductive surgery and the onset of sepsis, the severity of sepsis, antibiotic treatment, type of medical intervention, and ICU length of stay. Furthermore, follow-up data, i.e., recurrence status and date, survival status, cause and date of death, or last follow-up date, were collected. The cause of death was identified and was presumed cancer-related if the patient had advanced recurrent disease at time of death. If applicable, further data on patients’ recurrent EOC diagnosis and treatment were collected.

### 2.4. Oncologic Outcomes

Overall survival (OS) was defined as the time between the date of diagnosis and death or the last follow-up date (censoring date: 31 January 2022). Progressive or recurrent disease was defined as clinical signs of tumor growth, i.e., either increase in CA-125 serum levels (twice the upper limit of CA-125 serum level on two separate occasions at least one week apart) or tumor lesions visible on imaging (either growth of pre-existing or development of new lesions), combined with the clinical judgment of the treating medical oncologist or gynecologic oncologist. Progression-free survival (PFS) was defined as the time between the date of diagnosis and the date of disease progression or recurrence or the date of death, whichever occurred first. The last follow-up date, instead of date of death, was used to calculate the PFS and OS of patients who were still alive and did not experience progressive or recurrent disease.

### 2.5. Statistical Analysis

Patient, tumor, and treatment characteristics were summarized. Differences in clinicopathological characteristics between patients were assessed in a descriptive manner. The PFS and OS of patients were calculated. In addition, to correct for possible differences in case-mix, sepsis patients were compared to a propensity-score-matched group from the NCR in a sensitivity analysis. Patients were matched using 1:3 nearest neighbor matching on age, FIGO stage, histologic subtype, grade, treatment approach, bowel surgery, residual disease after debulking, and year of diagnosis. Survival curves using the Kaplan–Meier method were plotted to assess possible differences in OS and PFS between the sepsis patients (who comprised solely FIGO stage IIIC and IV EOC) and unmatched or matched FIGO stage IIIC and IV EOC patients from the NCR. In addition, Cox regression analyses were conducted to assess differences in OS and PFS between the sepsis and unmatched or matched patients from the NCR. The hazard ratios (HR) and their associated 95% confidence intervals (95%-CI) were reported. The Cox regression analysis was stratified by the matching group variable for the analysis using matched patients from the NCR. All statistical analyses were performed using STATA/SE, version 17 (Stata-Corp, College Station, TX, USA) and R, version 4.0.3 (http://www.r-project.org, accessed on 1 July 2022).

### 2.6. Ethical Approval

Ethical approval for this study was acquired from the Medical Ethical Committees (CMO 2019-5390) of all participating centers. Rules for obtaining informed consent were waived by the committees since additional privacy protection measures were taken to ensure that collected data were not traceable to individual patients. This study was carried out in accordance with the applicable rules concerning the review of research ethics committees and informed consent in the Netherlands.

## 3. Results

### 3.1. Study Population

**Figure 1:**
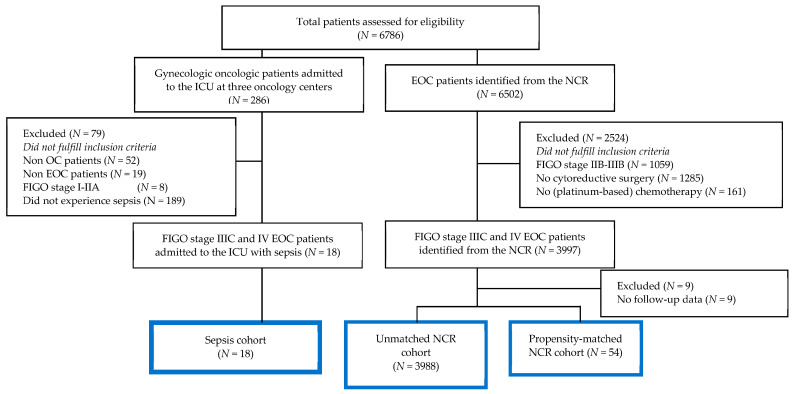
Flowchart of the study population (sepsis and NCR cohort).

A total of 215 patients with advanced-stage EOC were admitted to the ICU between 1 January 2006 and 1 January 2019 at one of the three hospitals. Among this group, 18 patients experienced sepsis with an abdominal focus (Figure 1). In addition, a total of 3988 FIGO stage IIIC and IV patients who underwent standard treatment for EOC were identified from the NCR (Figure 1). This group of advanced-stage EOC patients from the NCR was used as a control group. In an additional sensitivity analysis, a smaller subgroup of patients (N = 54) from the NCR was matched (1:3) based on prognostic factors and demographic characteristics (Figure 1).

### 3.2. Patient and Tumor Characteristics

Patient and tumor characteristics of the sepsis and NCR cohorts are summarized in Table 1. The median (IQR) age of the patients was 65 (60–73) years for the sepsis cohort. Thirteen patients had FIGO stage IIIC and five had FIGO stage IV EOC. Serous EOC type was the predominant histologic subtype (16/18). The sepsis patients underwent either primary cytoreductive surgery (PCS, 4/18) or interval cytoreductive surgery after neoadjuvant chemotherapy (NACT-ICS, 14/18). Similar proportions were observed in the unmatched NCR cohort. However, 13 sepsis patients (72%) underwent bowel surgery as part of cytoreductive surgery from the sepsis cohort, which was higher than the unmatched NCR cohort (815/3988, 21%). Five patients of the sepsis cohort did not receive adjuvant chemotherapy after interval debulking due to prior chemotherapy resistance (1/18), prolonged postoperative recovery (1/18), and postoperative mortality (3/18). All other sepsis patients completed their platinum-based chemotherapy as part of their primary EOC treatment. The differences in case-mix between the unmatched NCR cohort and the sepsis cohort were absent in the propensity-matched NCR cohort. Full details on patients’ EOC characteristics of the 18 sepsis patients are provided in Supplementary Table S3.

### 3.3. Sepsis Characteristics

The patients’ sepsis characteristics are summarized in Table 2. Most patients experienced sepsis after treatment for primary EOC (16/18), whereas two patients experienced sepsis after surgical treatment for recurrent disease. Sepsis occurred within 14 days of cytoreductive surgery for all patients except one (Patient E). Patient E developed urosepsis approximately five months after secondary cytoreductive surgery due to a blocked nephrostomy catheter that was inserted to manage a urinoma caused by an iatrogenic ureter injury. In most patients (13/18), sepsis was caused by bowel complications such as anastomotic leakage or bowel perforation. Four patients (Patients C, E, G, and O) developed sepsis due to a vaginal cuff abscess (leading to infective endocarditis), urosepsis, pancreatic fluid leakage, and gastric perforation, respectively. The exact site of infection was unclear for one patient (Patient F) since she experienced both pulmonary and abdominal complaints. All patients were treated with broad-spectrum antibiotics and underwent some type of source control, such as a relaparotomy (including bowel surgery and/or abscess drainage), or drainage. Most patients (15/18) recovered from sepsis, while three patients died from the complications of their sepsis during their hospital stay. Full details on patients’ sepsis characteristics are provided in Supplementary Table S4.

### 3.4. Oncologic Outcomes

**Figure 2:**
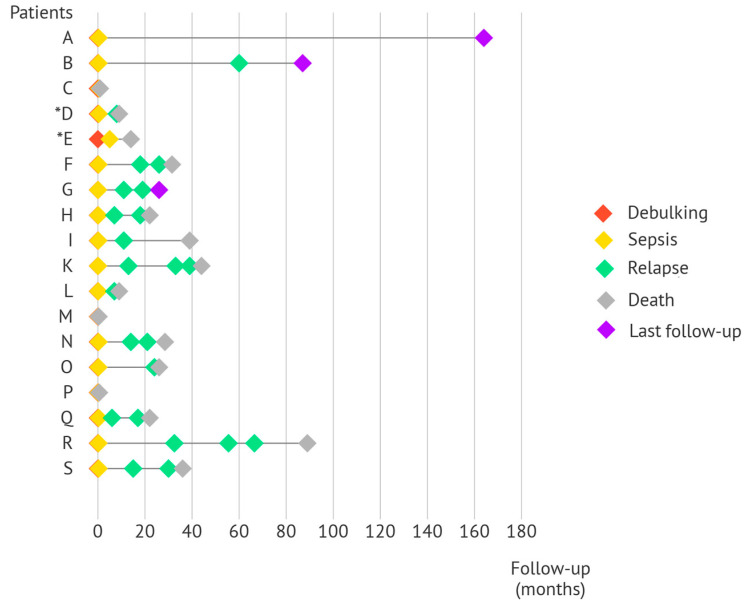
Timeline of the sepsis patients which outlines their oncologic outcomes. The timeline starts at the time of cytoreductive surgery. Sepsis occurred within a fortnight of cytoreductive surgery for 17/18 patients. As a result, the red diamond (indicative of cytoreductive surgery) is less noticeable for these patients. Patients C, M, and P died from the complications of sepsis. * Patients D and E experienced sepsis during treatment after cytoreductive surgery for first and second relapses, respectively.

Of the 15 patients who recovered from sepsis, 13 patients experienced sepsis during primary EOC treatment. Among them, only Patient A has not developed disease recurrence after finishing treatment more than 13 years ago. Other patients did develop progressive or recurrent disease often within a year after completing primary EOC treatment or later (Patients B, O, and R). The remaining two patients (Patients D and E) developed sepsis after secondary cytoreductive surgery in the treatment of EOC relapse. Both patients died within 18 months of treatment. The timeline of the different patients and their oncologic outcomes are demonstrated in Figure 2. Full details on patients’ oncologic outcomes are provided in Supplementary Table S5.

### 3.5. Survival Outcomes

**Figure 3:**
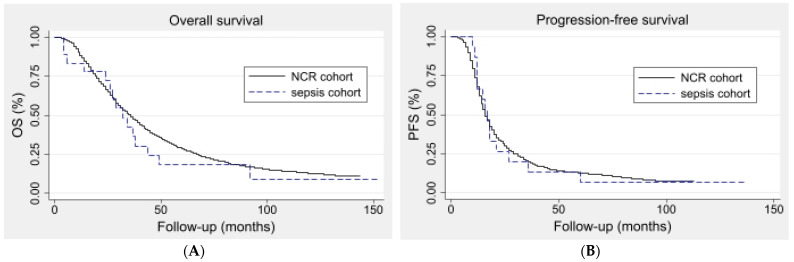

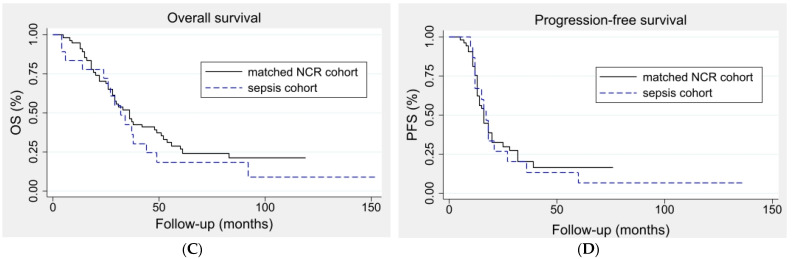
Kaplan–Meier curves of the sepsis and unmatched NCR cohort as well as the propensity-matched NCR cohort. The Kaplan–Meier curves of the OS of the sepsis cohort (N = 18) and unmatched NCR cohort (N = 3988) are depicted (**A**). The median (IQR) OS was 31 (24–44) months for the sepsis cohort and 35 (20–60) months for the NCR cohort (*p* = 0.56). Kaplan–Meier curves of the PFS of the sepsis cohort (N = 18) and unmatched NCR cohort (N = 3852) * are depicted (**B**). The median (IQR) PFS was 16 (11–21) and 16 (11–27) months, respectively (*p* = 0.90). * A total of 136 patients were further excluded from this analysis compared to Figure 1 and Figure 3 due to missing follow-up data regarding the patients’ recurrence status. The Kaplan–Meier curves of the OS of the sepsis cohort (N = 18) and 1:3 propensity-matched NCR cohort (N = 54) are depicted (**C**). The median (IQR) OS was 31 (24–44) months for the sepsis cohort and 36 (20–56) months for the NCR cohort (*p* = 0.40). Kaplan–Meier curves of the PFS of the sepsis cohort and propensity-matched NCR cohort are depicted (**D**). The median (IQR) PFS was 16 (11–21) and 16 (12–21) months, respectively (*p* = 0.72).

Figure 3A,B demonstrate the Kaplan–Meier survival curves used to calculate the median OS and PFS of the sepsis and unmatched NCR patients, respectively. The median (IQR) OS was 31 (24–44) months for the sepsis cohort compared to 35 (20–60) months for the unmatched NCR cohort (*p* = 0.56). The median (IQR) PFS was 16 (11–21) months for the sepsis cohort and 16 (11–27) months for the unmatched NCR cohort (*p* = 0.90). The Cox regression analyses demonstrated a HR of 1.16 (95%-CI 0.70–1.93) for the OS and a HR of 1.03 (95%-CI 0.61–1.75) for the PFS.

### 3.6. Sensitivity Analysis

**Figure 4:**
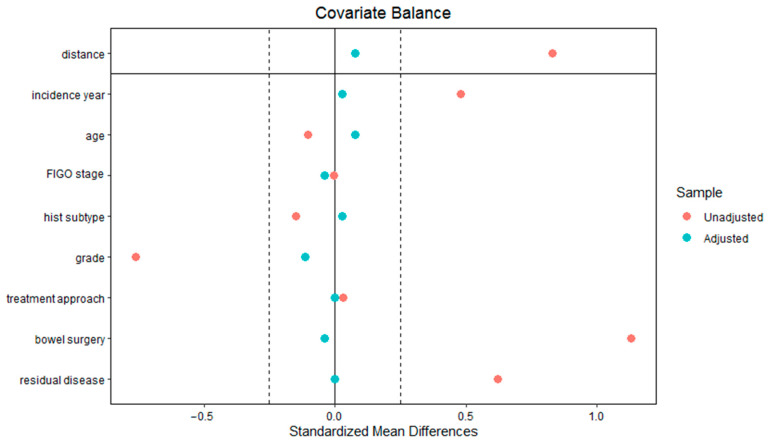
The Love plot demonstrates the absolute standardized mean difference of each variable in the unadjusted data (before propensity score matching) and adjusted data (after propensity score matching). The plot demonstrates that the covariates showed better balance in the adjusted data.

Figure 3C,D demonstrate the Kaplan–Meier estimates of the median OS and PFS of the sepsis and the propensity-matched NCR cohort, respectively. The median (IQR) OS was 31 (24–44) months for the sepsis cohort compared to 36 (20–56) months for the propensity-matched NCR cohort (*p* = 0.40). The median (IQR) PFS was 16 (11–21) months for the sepsis cohort and 16 (12–21) months for the propensity-matched NCR cohort (*p* = 0.72). The Cox regression analyses demonstrated a HR of 1.42 (95%-CI 0.72–2.80) for the OS and a HR of 1.01 (95%-CI 0.49–2.10) for the PFS. The results from the propensity score matching analysis are demonstrated in Figure 4.

## 4. Discussion

During sepsis, the initial activation of the immune response may potentially be beneficial, while sepsis-induced immunosuppression may theoretically also harm cancer patients. To investigate the interplay between sepsis and ovarian cancer, our multicenter observational study provides a descriptive analysis of advanced-stage EOC patients who experienced sepsis after primary or recurrent EOC diagnosis. Our data indicate that, overall, sepsis does not influence the prognosis of advanced-stage EOC patients in terms of progression-free and overall survival. Apart from our finding that 3/18 (~17%) of the advanced-stage EOC patients died from the complications of sepsis, consistent with the current in-hospital mortality rate of 20–40% of cancer patients with sepsis or septic shock [17,22], the development of sepsis, overall, did not benefit or harm EOC patients.

It is recognized that the immune response in sepsis can be characterized by the simultaneous activation of pro-inflammatory and anti-inflammatory processes [15,17]. Specifically, the initially dominant hyper-inflammatory response, also known as the ‘cytokine storm’, in the first few days is characterized by increased levels of tumor necrosis factor-alpha (TNF-α), interleukin-1 beta (IL-1β), and interleukin-6 (IL-6) [15]. These proinflammatory cytokines are generally responsible for immune response activation (i.e., IL-6), cytotoxic and cytostatic effects against cancer cells (i.e., TNF-α), and the recruitment and activation of immune cells along with other pro-inflammatory cytokines (i.e., IL-1β) [23]. Simultaneously, both the innate and adaptive immune systems will start to dampen this hyper-inflammatory phase. As a result, patients may undergo either a controlled anti-inflammatory response, which enables them to return to immune homeostasis, or an uncontrolled anti-inflammatory response, which may lead them into a sustained immunosuppressed phase [15]. These immune processes may be related to reported favorable and unfavorable oncologic outcomes of cancer patients who experience sepsis [12]. Thus, it might be possible that overall there is no effect, but a subgroup of patients does benefit, while another subgroup experiences harm of this variable immune response.

This notion is illustrated by observations that the hyper-inflammatory phase of sepsis exerts a negative effect on the antitumor-responsive capacity in tumor-bearing mice who were pre-exposed to chemotherapy prior to sepsis when compared to those who were not [15,24,25,26]. Conversely, upfront debulking in EOC was demonstrated to reduce circulating regulatory T-cells (Tregs) and to increase CD8+ T-cells function, which resulted in a surgically induced reduction of the immunosuppressive environment [4,27,28]. These changes in the immune cells and their function were not observed in patients who underwent neoadjuvant chemotherapy or in those with recurrent disease [27]. In line with this, sepsis patients who underwent neoadjuvant chemotherapy before undergoing cytoreductive surgery, or patients who experienced sepsis after cytoreductive surgery for recurrent disease in our study, did not demonstrate favorable oncologic or survival outcomes. Conversely, sepsis patients who underwent primary cytoreductive surgery comprised two patients who demonstrated prolonged EOC survival and one patient who did not. However, no definite conclusions can be drawn due to the small number of these patients.

Another concept is that sepsis-induced immunosuppression can be compartmentalized as shown in murine models [16,29]. Tissue-resident memory CD8 T cells that reside in non-lymphoid tissues, contrary to the circulatory CD8 T cells, do not experience a loss in numbers or function after sepsis, probably due to their secluded localization and the inability of produced cytokines to reach them [16,29].

In addition, injections of anaerobic bacteria administered in the intraperitoneal cavity demonstrated better tumor targeting than when administered intravenously in ovarian-cancer-bearing murine models [26]. Since EOC predominantly operates in the intraperitoneal cavity, this seems to be the localization where the antitumor immune response needs to happen. Consequently, it could be that a systemic inflammatory response, related to other sites of infection, was insufficiently able to target the tumor or elicit a strong antitumor response in some of our cases.

Nevertheless, it is important to note that, contrary to the aforementioned case report, most patients in our study underwent successful EOC treatment often leaving microscopic or no residual disease. In addition, EOC treatment often comprised adjuvant chemotherapy for the patients. Thus, it appears plausible that the immunosuppressive effects of chemotherapy might have diminished the antitumor-responsive capacity of sepsis in our patients. Hence, it remains unclear to what extent the patients could have benefitted from the pro-inflammatory response of sepsis and to what extent sepsis essentially impacted the patients’ oncologic or survival outcomes. Particularly, it is speculative whether sepsis could have induced an antitumor response in the two patients who demonstrated prolonged EOC survival or whether their increased survival was within normal variation or due to other favorable factors.

Certain limitations apply to our study. Our study was mainly limited by its observational nature and the small group of EOC patients admitted to the ICU. To ensure the inclusion of patients who experienced fulminant sepsis, patients were identified through ICU departments. Therefore, sepsis patients without an ICU admission will have been missed. In addition, the small group of patients comprised a heterogeneous group, which may have impacted the statistical power to detect an association between sepsis and EOC survival. As a result, no definite conclusions can be drawn from our study. In addition, it remains possible that the occurrence of sepsis could be beneficial in a selected group of advanced-stage EOC patients, while there may be a harmful effect in another subgroup. This may be related to different factors, such as the site of infection, the extent of the inflammatory response, and the timing of sepsis relative to EOC diagnosis. Furthermore, since oxidative stress participates in the pathogenesis of both sepsis as well as ovarian cancer, different interactions with endogenous and exogenous antioxidant defense systems in patients may also play a role [30,31,32]. Also, the impact of sepsis in EOC patients with high tumor burden may be different. Nevertheless, this is the first study to assess the impact of sepsis on advanced-stage EOC where detailed information was collected on patients’ EOC and sepsis. The use of nationwide data to compare differences in survival outcomes between the sepsis patients and the unmatched and propensity-matched patients from the NCR represents a strength of this study.

To further investigate the impact of sepsis (and the earlier mentioned aspects of timing, severity, and so forth) on the antitumor response in advanced-stage EOC and its impact on EOC growth and development, it would be interesting to explore this in EOC-bearing mice experiencing cecal ligation and puncture-induced sepsis compared to EOC-bearing mice in which sepsis was not induced. An experimental study might eventually lead to a new immunotherapeutic strategy for a specific group of EOC patients who did not receive prior treatment. Current ongoing clinical trials (e.g., FIRST trial, NCT03602859) so far focus on combining immunotherapy with chemotherapy, anti-angiogenesis drugs, immune checkpoint inhibitors, or other immunotherapies in an effort to enhance the antitumor activity of immunotherapeutic agents [33,34]. While other promising approaches such as chimeric antigen receptor (CAR)- and T-cell receptor (TCR)-engineered T cells, dendritic vaccinations, and oncolytic viruses are still emerging, response rates to immunotherapy remain modest among EOC patients [6,34] and our study adds to the notion that sepsis or the subsequent immune response does not substantially influence the prognosis of patients with EOC.

## 5. Conclusions

In conclusion, in our observational study, we found no indications that postoperative sepsis may affect cancer prognosis or survival for advanced-stage EOC patients.

## Figures and Tables

**Table 1:** EOC characteristics of the sepsis cohort (N = 18), NCR cohort (N = 3988), and propensity-matched NCR cohort (N = 54).

Characteristic	Sepsis Cohort (N = 18)	NCR Cohort (N = 3988)	Propensity-Matched NCR Cohort ^a^ (N = 54)
	Number of Patients (IQR) or (%)	Number of Patients (IQR) or (%)	Number of Patients (IQR) or (%)
Age at diagnosis (in years)			
Median	65 (60–73)	65 (20–88)	63 (43–79)
FIGO stage			
Stage IIIC	13 (72)	2884 (72)	38 (70)
Stage IV	5 (28)	1104 (28)	16 (30)
Histologic subtype			
Serous	16 (89)	3211 (80)	46 (85)
Mucinous	0 (0)	72 (2)	0 (0)
Endometrioid	0 (0)	124 (3)	5 (9)
Clear cell	0 (0)	104 (3)	0 (0)
Adenocarcinoma NOS	2 (11)	428 (11)	2 (6)
Other	0 (0)	49 (1)	0 (0)
Treatment approach			
PCS	4 (22)	1144 (29)	16 (30)
NACT-ICS	14 (78)	2844 (71)	38 (70)
Bowel surgery			
No	5 (28)	3075 (77)	13 (26)
Yes	13 (72)	851 (21)	40 (74)
Unknown	0 (0)	62 (2)	0 (0)
Residual disease after debulking			
0 cm	12 (67)	1939 (49)	38 (70)
≤1 cm	6 (33)	1487 (37)	14 (26)
>1 cm	0 (0)	500 (12)	2 (4)
Unknown	0 (0)	62 (2)	0 (0)
Chemotherapy (primary treatment)			
Yes, adjuvant	4 (22)	1144 (29)	16 (30)
Yes, neoadjuvant and adjuvant	9 (50)	2725 (68)	37 (68)
Yes, neoadjuvant only	5 (28)	119 (3)	1 (2)

Abbreviations: IQR, interquartile range; FIGO, International Federation of Gynecology and Obstetrics; PCS, primary cytoreductive surgery; NACT-ICS, neoadjuvant chemotherapy followed by interval cytoreductive surgery; NOS, not otherwise specified. ^a^ The patients were matched according to the following variables: year of diagnosis, age, FIGO stage, histologic subtype, grade, treatment approach, bowel surgery, and residual disease after debulking.

**Table 2:** Sepsis characteristics of the sepsis cohort (N = 18).

Characteristic	Number of Patients	(IQR) or %
Sepsis diagnosed during		
Primary EOC treatment	16	89%
Recurrent EOC treatment	2	11%
Time between surgery and sepsis onset (days)		
Median	7	(5–9)
Severity of sepsis		
Non-vasopressor-dependent sepsis	13	72%
Vasopressor-dependent sepsis	5	28%
Site of infection		
Bowel complications	13	72%
Gastric perforation	1	5.5%
Pancreatic fluid leakage	1	5.5%
Urosepsis	1	5.5%
Vaginal cuff abscess	1	5.5%
Unclear	1	5.5%
Source control		
Abscess drainage	3	17%
Laparotomy including abscess drainage	13	72%
Insertion nephrostomy catheter	1	5.5%
Mechanical ventilation	1	5.5%
Antibiotic treatment duration (days)		
Median	10	(6–10)
ICU length of stay		
Median	3	(1–8)
Hospital length of stay		
Median	24	(18–42)

## Data Availability

The data presented in this study are available in the Supplemental material.

## Supplementary Materials

The following supporting information can be downloaded at: https://www.mdpi.com/article/10.3390/cancers15184642/s1. Supplementary Table S1. Definitions of sepsis. Supplementary Table S2. Sequential Organ Failure Assessment (SOFA) Score System. Supplementary Table S3. EOC diagnosis and treatment characteristics of the study patients. Supplementary Table S4. Sepsis diagnosis and treatment characteristics of the study patients. Supplementary Table S5. Oncologic and survival outcomes data of the study patients.
