# Supplementary material for: Impact of Sepsis on the Oncologic Outcomes of Advanced Epithelial Ovarian Cancer Patients: A Multicenter Observational Study
Source: Cancers (Basel). 2023 Sep 20;15(18):4642. doi: 10.3390/cancers15184642 (PMC10526225; doi:10.3390/cancers15184642)
Supplement: Supplementary file 1 [file cancers-15-04642-s001.zip › cancers-2487489-SI.pdf]

Impact of Sepsis on the Oncologic Outcomes of Advanced Epithelial Ovarian Cancer Patients: A Multicenter Observational Study

S. A. Said [1,2], J. A. de Hullu [1], M. A. van der Aa [2], J. E.W. Walraven [3], R. L. M. Bekkers [4,5], B. F. M. Slangen [,5,6], P. Pickkers [7] and A. M. van Altena [1]

[1] Department of Obstetrics and Gynecology, Radboud Institute for Health Sciences, Radboud University Medical Center, 6525 EP Nijmegen, The Netherlands
[2] Department of Research and Development, Netherlands Comprehensive Cancer Organization (IKNL), 3511 DT Utrecht, The Netherlands
[3] Department of Medical Oncology, Radboud University Medical Center, 6525 EP Nijmegen, The Netherlands
[4] Department of Obstetrics and Gynecology, Catharina Hospital, 5623 EJ Eindhoven, The Netherlands
[5] GROW–School for Oncology and Reproduction, University of Maastricht, 6229 GT Maastricht, The Netherlands
[6] Department of Obstetrics and Gynecology, Maastricht University Medical Centre, 6229 HX Maastricht, The Netherlands
[7] Department of Intensive Care Medicine, Radboud University Medical Center, 6525 EP Nijmegen, The Netherlands

Supplementary

Supplementary Tables

Supplementary Table S1. Definitions of sepsis.

	Sepsis-2	Sepsis-3
SIRS	At least 2 of the following:<br>- temperature >38°C or <36°C,<br>- heart rate >90/min,<br>- respiratory rate >20/min or $PaCO_2$ <32mmHg,<br>- white cell count >12000 or 4000 or >10% immature (bands) forms	Not applicable
SOFA (see table 2)	Not applicable	Respiratory (PaO_2/FiO_2),<br>Nervous (Glasgow coma scale), Cardiovascular (mean arterial pressure or vasopressor),<br>Liver (bilirubin),<br>Coagulation (platelets),<br>Renal (creatinine)
Sepsis	SIRS + suspected infection	Increase in SOFA score $\geq$ 2 points + suspected infection
Septic shock	SBP <90mmHg, reduction in SBP$\geq$ 40mmHg from baseline, or MAP <60mmHg despite fluid resuscitation	Vasopressors to maintain MAP $\geq$ 65mmHg and lactate $\geq$ 2mmol/dL despite adequate volume resuscitation

Abbreviations: SIRS, systemic inflammatory response syndrome; SOFA, sequential organ failure assessment; SBP, systolic blood pressure; MAP, mean arterial pressure.

Supplementary Table S2. Sequential Organ Failure Assessment (SOFA) Score System

	**Score**				
**System**	**0**	**1**	**2**	**3**	**4**
Respiration					
PaO_2/FIO_2, mmHg (kPa)	≥400 (53.3)	<400 (53.3)	<300 (40)	<200 (26.7) with respiratory support	<100 (13.3) with respiratory support
Coagulation					
Platelets,×10^3/μL	≥150	<150	<100	<50	<20
Liver					
Bilirubin, mg/dL (μmol/L)	<1.2 (20)	1.2–1.9 (20–32)	2.0–5.9 (33–101)	6.0–11.9 (102–204)	>12.0 (204)
Cardiovascular					
Mean arterial pressure or vasopressor	MAP ≥70 mm Hg	MAP <70 mm Hg	Dopamine <5 or dobutamine (any dose)	Dopamine 5.1–15 or epinephrine ≤0.1 or norepinephrine ≤0.1	Dopamine >15 or epinephrine >0.1 or norepinephrine >0.1
Central nervous system					
Glasgow Coma Scale (score)	15	13–14	10–12	6–9	<6
Renal					
Creatinine, mg/dL (μmol/L)	<1.2 (110)	1.2–1.9 (110–170)	2.0–3.4 (171–299)	3.5–4.9 (300–440)	>5.0 (440)
Urine output, mL/d				<500	<200

Abbreviations: FIO2, fraction of inspired oxygen; MAP, mean arterial pressure; PaO2, partial pressure of oxygen.

Supplementary Table S3. EOC diagnosis and treatment characteristics of the study patients

Patients	Age	Inci-dence date	FIGO stage	Histologic sub-type	Tumor grade	Treatment approach	Bowel surgery	Type of bowel surgery	Residual disease	Type of chemotherapy	Completion of chemotherapy	Response of chemotherapy
**Patient A**	68	2008	IV	Serous	Grade 3	PCS	Yes	Ileocaecal resection, rectosig-moid resection with a construc-tion of an end colostomy.	0 cm	Adjuvant 6 cycles of car-boplatin/ paclitaxel	Yes	Complete re-mission
**Patient B**	68	2014	IIIC	Serous	Grade 3	PCS	Yes	Tumor deposits were removed from mesentery of the colon as-cendens	0 cm	Adjuvant 6 cycles of car-boplatin/ paclitaxel	Yes	Complete re-mission
**Patient C**	75	2015	IIIC	Serous	Grade 3	NACT-ICS	No	-	< 1 cm	Neoadjuvant 3 cycles of carboplatin/ paclitaxel	No (patient died)	-
**Patient D**	32	2009	IIIC	Serous	Grade 1	NACT-ICS	Yes	Resection of terminal ileum, rec-tosigmoid resection with con-struction of a temporary colos-tomy.	0 cm	Neoadjuvant 3 cycles of carboplatin/ paclitaxel	No (due to chem-otherapy re-sistance)	Progressive dis-ease (during ne-oadjuvant chemo)
**Patient E**	40	2009	IIIC	Serous	Grade 2	PCS	Yes	Appendectomy	< 1 cm	Adjuvant 6 cycles of car-boplatin/ paclitaxel	Yes	Complete re-mission
**Patient F**	60	2011	IIIC	Serous	Grade 2	NACT-ICS	Yes	Right hemicolectomy, en bloc partial ileum and rectosigmoid resection and construction of a temporary colostomy.	0 cm	Neoadjuvant 3 cycles of carboplatin/ paclitaxel<br><br>OVHIPEC with cisplatin<br><br>Adjuvant 3 cycles of car-boplatin	Yes (no adjuvant paclitaxel given due to adverse re-actions)	Complete re-mission
**Patient G**	61	2019	IIIC	Serous	Grade 3	NACT-ICS	No	(Splenectomy)	< 1 cm	Neoadjuvant 2 cycles of carboplatin/paclitaxel; then Neoadjuvant 6 cy-cles of dose-dense car-boplatin/paclitaxel<br><br>OVHIPEC with cisplatin	Yes	Partial remis-sion

										Adjuvant 2 cycles of dose-dense carboplatin/paclitaxel		
**Patient H**	73	2016	IVA	Serous	Grade 3	NACT-ICS	Yes	A serosa injury of the colon transversum was sutured; no other bowel surgery was performed.	<1 cm	Neoadjuvant 6 cycles of carboplatin/ paclitaxel<br><br>Adjuvant 3 cycles of carboplatin/ paclitaxel	Yes	Stable disease
**Patient I**	73	2015	IV	Serous	Grade 3	NACT-ICS	No	-	0 cm	Neoadjuvant 6 cycles of carboplatin/paclitaxel<br><br>Adjuvant 3 cycles of carboplatin/ paclitaxel	Yes	Complete remission
**Patient K**	80	2015	IIIC	Serous	Grade 3	NACT-ICS	Yes	Tumor deposits were removed from mesentery of the colon transversum	0 cm	Neoadjuvant 3 cycles of carboplatin/paclitaxel<br><br>Adjuvant 3 cycles of carboplatin/ paclitaxel	Yes	Complete remission
**Patient L**	64	2015	IIIC	Serous	Grade 3	NACT-ICS	Yes	Lower anterior resection and extended right hemicolectomy and ileal resection with an end-to-end anastomosis; appendectomy	<1 cm	Neoadjuvant 6 cycles of carboplatin/ paclitaxel<br><br>Adjuvant 3 cycles of carboplatin/ paclitaxel	Yes	Complete remission
**Patient M**	70	2016	IIIC	Serous	Grade 3	NACT-ICS	Yes	Bowel surgery performed: reversal of ileostomy; side to side anastomosis	0 cm	Neoadjuvant 3 cycles of carboplatin/ paclitaxel	No (patient died)	-

**Patient N**	64	2011	IIIC	Adenocarcinoma NOS	Unknown	NACT-ICS	No	-	0 cm	Neoadjuvant 3 cycles of carboplatin/ paclitaxel<br><br>Adjuvant 3 cycles of carboplatin. Followed by 8 cycles of bevacizumab/ cyclofosfamide	Yes	Complete remission
**Patient O**	66	2008	IVB	Adenocarcinoma NOS	Unknown	NACT-ICS	No	-	0 cm	Neoadjuvant 3 cycles of carboplatin/ paclitaxel	No (due to postoperative complications)	Complete remission
**Patient P**	67	2019	IIIC	Serous	Grade 3	NACT-ICS	Yes	Tumor deposits were removed from mesentery; no other bowel surgery was performed.	<1 cm	Neoadjuvant 3 cycles of carboplatin/ paclitaxel	No (patient died)	-
**Patient Q**	64	2015	IIIC	Serous	Grade 3	NACT-ICS	Yes	Ileocecal resection (side-to-side anastomosis) and lower anterior resection with the creation of end colostomy.	0 cm	Neoadjuvant 3 cycles of carboplatin/ paclitaxel<br><br>OVHIPEC (cisplatin)<br><br>Adjuvant 3 cycles of carboplatin/ paclitaxel	Yes	Complete remission
**Patient R**	48	2011	IVA	Serous	Grade 3	NACT-ICS	Yes	Excisions of multiple lesions on the sigmoid and ascending colon (primary closure of the wounds).	0 cm	Neoadjuvant 3 cycles of carboplatin/ paclitaxel<br><br>Adjuvant 4 cycles of carboplatin/ paclitaxel	Yes	Complete remission
**Patient S**	61	2012	IIIC	Serous	Grade 3	PCS	Yes	Sigmoid resection and ileocecal resection (side-to-end anastomosis colon transversum and ileum).	0 cm	Adjuvant 6 cycles of carboplatin/ paclitaxel	Yes	Complete remission

Supplementary Table S4. Sepsis diagnosis and treatment characteristics of the study patients

	ICU admission for sepsis	Sepsis after primary treatment	Time (days) between surgery sepsis	Cause of sepsis	Severity of sepsis	Antibiotic treatment for sepsis	Bacterial culture obtained	Time (days) between sepsis and intervention	Type of intervention	Treatment response (for sepsis)	Length-of-stay ICU	Length-of-stay hospital
**Patient A**	Yes	Yes	9	Anastomotic leakage of the colorectal anastomosis (no bowel ischemia)	Non-vasopressor dependent sepsis	10 days<br><br>Piperacillin/ Tazobactam (2 days);<br><br>Switch to Meropenem (8 days)	Yes, Multidrug-resistant E. coli (peritoneal fluid)	0	Relaparotomy; cleaning out of abscess and construction of a double loop ileostomy	Full recovery	1	26
**Patient B**	Yes	Yes	7	Bowel perforation at the distal site of the ascending colon (no bowel ischemia)	Non-vasopressor dependent sepsis	8 days<br><br>Piperacilin/ Tazobactam (7 days);<br><br>Switch to Ciprofloxacin (1 day)	Yes, no bacteria found in pleural fluid and blood culture.<br><br>Not determined for the peritoneal fluid	1	Relaparotomy; lavage of the abdominal cavity; right hemicolectomy and construction of an end ileostomy.	Full recovery	1	18
**Patient C**	Yes	Yes	14	Abscess at vagina cuff; followed by persistent sepsis due to infective mitral valve endocarditis	Vasopressor dependent sepsis (septic shock)	23 days<br><br>Piperacillin/ Tazobactam (7 days);<br><br>Switch to Teicoplanin/ Metronidazole (17 days)	Yes, Proteus Mirabilis (blood culture)<br><br>Later Multidrug-resistant E. coli and Enterococcus faecium (debris of vagina top)	3	Abscess drainage of the cuff of vagina	No recovery; (patient died at the ICU)	4 days (1st ICU admission for sepsis)<br><br>4 days (2nd ICU admission for sepsis	44
**Patient D**	Yes	No (after surgery for 1st relapse)	11	Ileal anastomotic leakage of the 2nd	Vasopressor dependent sepsis	>10 days<br><br>Piperacillin/ Tazobactam	Yes, Multidrug-resistant E. coli (peritoneal fluid)	0	1st intervention: relaparotomy; Vorlagerung procedure** and	Delayed recovery (2nd intervention	4	31

				anastomosis of the ileum					placing abdominal drains.<br><br>2nd intervention: operative drainage peritoneal fluid	was needed))		
**Patient E**	Yes	No (after surgery for 2nd relapse)	>150	Urosepsis due to a blocked nephrostomy catheter and urinoma	Non-vasopressor dependent sepsis	>10 days<br><br>Ceftriaxone (1 day);<br><br>Switch to Amoxicillin/Clavulanic acid	Yes, Multidrug-resistant E. coli (urine)	0	Insertion of a new nefrostomy catheter	Full recovery	1	63
**Patient F**	Yes	Yes	7	Bacterial translocation due to ileus or pneumosepsis due to aspiration pneumonia (pneumosepsis more likely)	Vasopressor dependent sepsis	5 days<br><br>Ceftazidime/ Metronidazole (5 days both)	Yes, Enterococcus faecium (peritoneal fluid collected from abdominal drain)	0	Intubation for mechanical ventilation<br><br>No surgical intervention was needed	Delayed recovery (after mechanical ventilation at the ICU)	9	42
**Patient G**	No (postop ICU for OVHIPEC)	Yes	2	Pancreatic fluid leakage which intraabdominal fluid collections	Non-vasopressor dependent sepsis	7 days<br><br>Ceftriaxone/ Metronidazole (1,5 days)<br><br>Switch to Piperacillin/Tazobactam (5 days both)	Yes, S. epidermidis (blood culture -> possible contamination)	1	Insertion of drain for pancreatic fluid drainage	Delayed recovery (after long-term drainage of the pancreatic fluid)	0 days (for sepsis)	10
**Patient H**	Yes	Yes	4	Bowel perforation colon transversum; followed by intra-abdominal abscess 10 days	Non-vasopressor dependent sepsis	5 days<br><br>Cefuroxime/ Metronidazole (5 days both)	Yes, Pseudomonas aeruginosa and Enterococcus faecium (peritoneal fluid)	0	1st intervention: Relaparotomy; considered to segment resection with the creation of an ostomy	Delayed recovery (2nd intervention was needed)	2	18

				after relaparotomy					(however refrained from performing this) and sutured the defect<br><br>2nd intervention: Ultrasound guided drainage intra-abdominal abscess			
**Patient I**	Yes	Yes	5	Bowel perforation of the rectosigmoid colon	Non-vasopressor dependent sepsis	6 days<br><br>Ceftriaxone/ Metronidazole (6 days both)	Yes, Gram-negative rods (not further determined) combined with anaerobic gut flora<br>(peritoneal fluid)	0	1st intervention: Relaparotomy (defect of rectosigmoid was sutured; creation of double loop ostomy of colon transversum)<br><br>2nd intervention: Sedation on ICU (4 days)	Delayed recovery (2nd intervention was needed)	4	18
**Patient K**	Yes	Yes	2	Bowel perforation jejunum	Non-vasopressor dependent sepsis	11 days<br><br>Ceftriaxone/ Metronidazole (5 days both)<br><br>Switch to Cefuroxime/ Metronidazole (6 days both)	Yes, anaerobic gut flora<br>(peritoneal fluid)	0	1st intervention: Relaparotomy (suturing of jejunum defect)<br><br>2nd intervention: Platzbauch repaired with Mesh	Delayed recovery (2nd surgical reintervention needed for Platzbauch)	7 (after 1st intervention)<br><br>4 (after 2nd intervention)	29
**Patient L**	Yes	Yes	4	Anastomotic leakage of the ileotransverso colon anastomosis (bowel ischemia)	Non-vasopressor dependent sepsis	4 days<br><br>Cefotaxime/ Metronidazole (4 days both)	Yes, Bacteroides fragilis<br>(blood culture)<br><br>Staphylococcus aureus combined with anaerobic	0	Relaparotomy (creation of ileotransversoostomy)	Full recovery	2	13

							flora (pus and wound fluid)					
**Patient M**	Yes	Yes	6	Bowel ischemia and anastomotic leakage of the sigmoid colon	Vasopressor-dependent sepsis	5 days<br><br>Cefuroxim/ Metronidazole (1 day both)<br><br>Switch to Cefotaxim/ SDD (1 day both)<br><br>Switch to Meroponem/ Metronidazole/ Vancomycin (3 days both)	Yes, Pseudomonas aeruginosa and anaerobic flora (peritoneal fluid)	0	1st intervention: Relaparotomy; drainage of fecal fluid in abdomen. The site of anastomotic leak or bowel ischemia found. Closed with abthera.<br><br>2nd intervention: Relaparotomy; resection of part of sigmoid with the anastomotic leakage; creation of colostomy	No recovery; (patient died at the ICU)	4	11
**Patient N**	Yes	Yes	8	Bowel perforation of the sigmoid colon as result of bowel ischemia	Non-vasopressor dependent sepsis	10 days<br><br>Cefuroxim/ Metronidazole/ Gentamycin	Yes, E. coli and Bacteroides fragilis (blood culture)<br><br>Bacteroides fragilis (peritoneal fluid)	0	1st intervention: Relaparotomy, drainage fecal fluid and creation of end colostomy<br><br>2nd intervention: Ultrasound guided drainage of abdominal fluid	Delayed recovery (2nd intervention was needed)	8	49
**Patient O**	Yes	Yes	7	Suspected gastric perforation (CT-imaging demonstrated signs of perforation. Even though the site	Non-vasopressor dependent sepsis	9 days<br><br>Cefuroxime/ Metronidazole (4 days both)	Yes, E. coli and Staphylococcus aureus (pus and wound)<br><br>Enterobacter cloaca,	0	1st intervention: Relaparotomy (no perforation of the colon found or fecal fluid in the abdomen but yellow pus)	Delayed recovery (2nd intervention was needed)	3 (after 1st intervention)<br><br>2 (after 2nd intervention)	60 (transferred to another hospital)

				of perforation was not found, an infiltrate was observed near antrum of the stomach)		Switch to Piperacillin/ Tazobactam/ Vancomycin (5 days both)	Enterococcus faecium and Candida albicans (peritoneal fluid)		2nd intervention: Relaparotomy (insertion of drains for the infiltrate around the antrum of the stomach)			
**Patient P**	Yes	Yes	6	Bowel ischemia of the colon transversum	Vasopressor-dependent sepsis	>10 days<br><br>Piperacilin/ Tazobactam/ Anidulgafungin<br>(1 day)<br><br>Switch to Cefotaxime/ Metronidazole<br>(6 days both)<br><br>Switch to Meropenem/ Vancomycin/ Anidulafungin/ SDD (selective digestive decontamination) (>3 days)	Yes, Enterococcus faecalis (peritoneal fluid and ascites)	5	Relaparotomy; creation of end colostomy; drainage of fecal fluid	No recovery; (patient died at the ICU)	15	16
**Patient Q**	No (post-op ICU due to OVHIPEC)	Yes	9	Spontaneous bacterial peritonitis (no anastomotic leakage or signs of bowel ischemia or bowel perforation)	Non vasopressor-dependent sepsis	10 days<br><br>Cefuroxim/ Metronidazole (10 days both)	No bacteria found in drain fluid (peritoneal fluid)	0	1st and 2nd intervention: Ultrasound guided abdominal drainage (excessive peritoneal fluid and no fecal fluid)	Delayed recovery (2nd US guided drainage was needed)	0	18
**Patient R**	Yes	Yes	7	Bowel perforation caecum or colon ascendens	Non vasopressor-dependent sepsis	10 days<br><br>Cefuroxim/ Metronidazole (10 days both)	Yes, Enterococcus faecalis and Escherichia coli (peritoneal fluid)	1	1st intervention: Relaparotomy comprising right hemicolectomy with the creation	Delayed recovery (patient needed CT-drainage	1	22

									of end-ileostomy and mucus fistula of the colon transversum (split stoma)<br><br>2nd intervention : CT-guided abdominal drainage	for paracolic abscess)		
**Patient S**	Yes	Yes	10	Bowel ischemia which caused a bowel perforation and leakage of fecal fluid of the colon	Non vasopressor-dependent sepsis	14 days<br><br>Gentamycin (1 dose)<br>Piperacilin/ Tazobactam (5 days both)<br><br>Switch to Amoxicilin/ Clavulanic acid<br>(2 days both)<br><br>Swith back to Piperacilin/Tazobactam (7 days both)	Yes, Escherichia coli<br>(peritoneal fluid)	0	1st intervention: Relaparotomy with creation of colostomy<br><br>2nd intervention: VAC-system	Delayed recovery (patient needed multiple washings and a VAC system for a wound infection)	1	31

Supplementary Table S5. Oncologic and survival outcomes data of the study patients

	**EOC recurrence**	**PFI* (months)**<br><br>**(last FU date if no recurrence)**	**Secondary cytoreductive surgery**	**Residual disease after secondary surgery**	**Chemotherapy for EOC recurrence**	**Type of chemotherapy for recurrence**	**Completion of chemotherapy for recurrence**	**Response after chemotherapy for recurrence**	**Death**	**Cause of death**	**OS (months)**	**PFS (months)**
**Patient A**	No	131	-	-	-	-	-	-	No	-	153	137
**Patient B**	Yes	56	No	-	Yes	1 cycle of carboplatin/paclitaxel;	Yes	Complete remission	No	-	85	60

						Switch to 5 cycles carboplatin/gem-citabine (No paclitaxel due to neu-ropathy)						
**Patient C**	*NA*	-	-	-	-	-	-	-	Yes	Septic shock**	-	-
**Patient D**	Yes	1st rec: 9 months<br><br>2nd relapse: 6 months	Yes (for 1st rec)	<1 cm	Yes	1st rec: 3 cycles of doxorubicin (Caelyx)<br><br>2nd relapse: none	No (patient wish to discontinue chemo-therapy)	1st rec: Progression of disease	Yes	Cancer related	24	PFS1: 12 months<br><br>PFS2: 11 months
**Patient E**	Yes	1st rec: 7 months<br><br>2nd relapse: 3 months	Yes (for 2nd rec)	0 cm	Yes	1st rec: 6 cycles of car-boplatin/paclitaxel<br><br>2nd relapse: None	Yes	1st rec: Complete remission<br><br>2nd relapse: Com-plete remission	Yes	Cancer related	37	PFS1: 12 months<br><br>PFS2: 7 months<br><br>PFS3: 18 months
**Patient F**	Yes	1st rec: 13 months<br><br>2nd relapse: 3 months	No	-	Yes	1st rec: 6 cycles of carboplatin<br><br>2nd relapse: None	No (patient refusal after 2nd relapse)	1st rec: Stable dis-ease for 3 months then Progressive disease	Yes	Cancer related	35	21
**Patient G**	Yes	1st rec: 8 months<br><br>2nd relapse: 4 months	No	-	Yes	1st rec: 6 cycles of carboplatin/ gemcitabine<br><br>Switch to maintenance therapy with Niraparib (PARP inhibitor)<br><br>2nd relapse: 3 cycles doxorubicin/ bevacizumab	Yes	1st rec: Stable dis-ease for 4 months then Progressive disease	No	-	27	15
**Patient H**	Yes	1st rec: 4 months	No	-	Yes	1st rec: 1 cycles of liposomal Doxo-rubicin and 2 cycles of paclitaxel and bevacizumab	Yes (patient died during chemotherapy for 2nd progression)	1st rec: Stable dis-ease	Yes	Cancer related	27	13

		2nd relapse: 3 months				Switch to 6 cycles of carboplatin/paclitaxel<br><br>2nd relapse: 2 cycles of gemcitabine						
**Patient I**	Yes	7.5 months	No	-	Yes	1 cycle of carboplatin/ paclitaxel	No (patient wish to discontinue)	Progressive disease	Yes	Cancer related	44	17
**Patient K**	Yes	1st rec: 9 months<br><br>2nd relapse: 6 months	No	-	Yes	1st rec: maintenance therapy of carboplatin/ cyclofosfamide<br><br>2nd relapse: 6 cycles of Doxorubicin Switch to maintenance therapy with tamoxifen	Yes	1st rec: Partial remission<br><br>2nd relapse: Progressive disease	Yes	Cancer related	48	17
**Patient L**	Yes	3	No	-	Yes	1 cycle of doxorubicin (Caelyx)	No (patient wish to discontinue)	Progressive disease	Yes	Cancer related	14	12
**Patient M**	*NA*	-	-	-	-	-	-	-	Yes	Septic shock**	6	-
**Patient N**	Yes	1st rec: 7 months<br><br>2nd relapse: 4 months	No	-	Yes	1st rec: 4 cycles of carboplatin/ paclitaxel<br><br>2nd relapse: 3 cycles of doxorubicin (Caelyx)	Yes	1st rec: Progressive disease<br><br>2nd relapse: Progressive disease	Yes	Cancer related	32	17
**Patient O**	Yes	1st rec: 23 months	No	-	Yes	Not reported	Not reported	Not reported	Yes	Cancer related	29	24
**Patient P**	*NA*	-	-	-	-	-	-	-	Yes	Septic shock**	4	-
**Patient Q**	Yes	1st rec: 6 months<br><br>2nd relapse: 11 months	No	-	Yes	1st rec: 6 cycles carboplatin/ paclitaxel (3 months after recurrence)<br><br>2nd relapse: 1 cycle of doxorubicin (Caelyx)<br>Palliative radiotherapy for necrotizing mass vagina cuff	No (patient refusal after 2nd relapse)	1st rec: Stable disease<br><br>2nd relapse: Progressive disease	Yes	Cancer related	26	10
**Patient R**	Yes	1st rec: 28 months	No	-	Yes	1st rec: 6 cycles carboplatin/ liposomal doxorubicin	No (carboplatin discontinued at 3rd	1st rec: Complete remission	Yes	Cancer related	93	36

		2nd relapse: 18 months<br><br>3rd relapse: 11 months				2nd relapse: 6 cycles of carboplatin /gemcitabine<br><br>3rd relapse: maintenance therapy with tamoxifen; followed by 4 cycles of carboplatin/ doxorubicin	relapse due to allergic reaction)	2nd relapse: Complete remission<br><br>3rd relapse: Progressive disease				
**Patient S**	Yes	1st rec: 15 months<br><br>2nd relapse: 15 months	No	-	Yes	1st rec: tamoxifen -> Progressive disease then start 6 cycles carboplatin/ paclitaxel<br><br>2nd relapse: 6 cycles of carboplatin/ doxorubicin (Caelyx)	Yes	1st rec: Partial remission<br><br>2nd relapse: Stable disease	Yes	Cancer related	38	17

*Platinum-free interval (PFI) was defined as the time between the date of receiving the last chemotherapy dose and the date of progressive or recurrent disease, or the date of death.

** Sepsis deaths
Three of the eighteen patients (Patients C, M, and P) died from the complications of a septic shock during their hospital admission. Patient C developed sepsis due to a vaginal cuff abscess which caused a fulminant infective endocarditis leading to septic shock and ultimately death. Patient M developed sepsis from an anastomotic leak. Although CT imaging showed signs of anastomotic leakage, no anastomotic leak was observed during an initial relaparotomy procedure. The following day, the patient's condition deteriorated rapidly. During a second relaparotomy procedure the site of anastomotic leak and ischemia of the colon was detected and managed with a sigmoid resection and colostomy. Nevertheless, she developed a septic shock leading to her death Lastly, Patient P developed sepsis without any clear signs of anastomotic leakage or bowel perforation on CT-imaging. The sepsis was initially managed with broad spectrum antibiotics. However, the patient's health worsened after which a colonoscopy confirmed bowel ischemia. She underwent a relaparotomy with bowel resection and creation of a colostomy. Nonetheless, the patient's sepsis did not respond to further treatment leading to septic shock and ultimately death.
